# „Deprescribing“ von Antipsychotika bei Demenz: Handlungsempfehlungen zu Dosisreduktion und Absetzversuchen

**DOI:** 10.1007/s00115-022-01343-w

**Published:** 2022-06-30

**Authors:** Carola Roßmeier, Jennifer Gast, Lina Riedl, Julia Hartmann, Sarah Kohl, Florentine Hartmann, Janine Diehl-Schmid

**Affiliations:** 1 Fakultät für Medizin, Klinik und Poliklinik für Psychiatrie und Psychotherapie, Klinikum rechts der Isar, Technische Universität München, München, Deutschland; 2 Fachbereich Alterspsychiatrie, kbo-Inn-Salzach-Klinikum GmbH, Gabersee 7, 83512 Wasserburg am Inn, Deutschland

**Keywords:** Psychische und Verhaltenssymptome bei Demenz, Antipsychotikabehandlung bei Demenz, Reduktion und Ausschleichen, Onlineumfrage, Konsens, Behavioral and psychological symptoms of dementia, Antipsychotics in dementia, Tapering, Online survey, Consensus

## Abstract

**Hintergrund:**

In Leitlinien zur Behandlung von psychischen und Verhaltenssymptomen bei Demenz („Behavioral and Psychological Symptoms of Dementia“ [BPSD]) wird empfohlen, Antipsychotika im Falle einer notwendigen Therapie zeitlich begrenzt und in möglichst geringer Dosis einzusetzen.

**Fragestellung:**

Um das Bewusstsein für die Risiken einer Antipsychotikabehandlung bei Patient:innen mit Demenz und die dadurch begründete Notwendigkeit einer zeitlich begrenzten und möglichst niedrig dosierten Antipsychotikabehandlung zu schärfen, wurden Handlungsempfehlungen zum Deprescribing von Antipsychotika bei Menschen mit Demenz entwickelt.

**Material und Methoden:**

Die Entwicklung der Handlungsempfehlungen erfolgte in einem mehrstufigen Prozess: 1. Literaturrecherche unter besonderer Würdigung von konsentierten Leitlinienempfehlungen und Ergebnissen einer eigenen Interventionsstudie (READY-Studie), 2. Diskussion und Konsentierung in einem Expert:innengremium, 3. Onlineumfrage von in Deutschland tätigen ärztlichen Demenzexpert:innen.

**Ergebnisse:**

Aus 51 im ersten Entwurf zusammengestellten Aussagen wurden nach der Diskussion im multiprofessionellen Expert:innengremium 32 Aussagen zum „Deprescribing“ (Reduktion und Ausschleichen) von Antipsychotika formuliert. 25 der 32 Aussagen wurden nach Konsentierung in einer Onlineumfrage durch ärztliche Demenzexpert:innen final übernommen.

**Diskussion:**

In Deutschland gibt es bislang keine konkreten Empfehlungen zur Vorgehensweise bei Reduktion und Absetzen von Antipsychotika, die zur Behandlung von BPSD bei Menschen mit Demenz eingesetzt werden. Die vorgelegte Arbeit soll als Handlungsempfehlung für Haus- und Fachärzt:innen dienen. Bei den Empfehlungen handelt es sich um keine Leitlinien, sie ersetzen nicht die Eigenverantwortlichkeit der Behandelnden und das individuell notwendige Vorgehen in Abhängigkeit von der Behandlungssituation.

**Zusatzmaterial online:**

Die Onlineversion dieses Beitrags (10.1007/s00115-022-01343-w) enthält zusätzliches Material. Beitrag und Zusatzmaterial stehen Ihnen auf www.springermedizin.de zur Verfügung. Bitte geben Sie dort den Beitragstitel in die Suche ein, die Videos finden Sie beim Beitrag unter „Ergänzende Inhalte“.

## Abstract (en)

**Background:**

Guidelines for the treatment of behavioral and psychological symptoms of dementia (BPSD) recommend that if antipsychotics are necessary, these should be used only for a limited time and with the lowest possible dose.

**Objective:**

In order to raise awareness of the risks of antipsychotic treatment in patients with dementia and the resulting need for time-limited and as low-dose antipsychotic treatment as possible, recommendations for deprescribing of antipsychotics in people with dementia have been developed.

**Material and methods:**

The recommendations were developed in a multistage process: 1. Literature search with special appreciation of agreed guideline recommendations and results of an intervention study (READY study) 2. Discussion and consensus in a panel of experts, 3. Online survey of medical dementia experts working in Germany.

**Results:**

From the 51 statements that were initially drafted 32 statements on antipsychotic deprescribing were formulated after the discussion in the multiprofessional expert panel. Of the 32 statements 25 were finally implemented after agreement by the experts in the online survey.

**Discussion:**

In Germany, there are so far no specific recommendations for the reduction and discontinuation of antipsychotics used to treat BPSD in people with dementia. The article is intended as recommendations for general practitioners and specialists. The recommendations are not guidelines and do not replace personal responsibility. The individual approach always depends on the individual treatment situation.

## Hintergrund

Im Verlauf einer Demenzerkrankung treten bei bis zu 90 % der Betroffenen psychische und Verhaltenssymptome („Behavioural and Psychological Symptoms of Dementia“ [BPSD]) wie Aggressivität, Erregung, Unruhe, Ängste oder auch Apathie, wahnhafte Symptome und Halluzinationen auf [24]. Die Ursachen hierfür sind vielfältig. Es besteht Konsens, dass BPSD primär durch sorgfältige Analyse und Beseitigung der Ursachen (z. B. Umgebungsfaktoren oder Schmerzen) und andere nichtmedikamentöse Maßnahmen behandelt werden sollen [1, 6, 11, 19, 21, 23, 27]. Dennoch werden Antipsychotika in dieser Indikation häufig eingesetzt. So wurden beispielsweise in Deutschland laut AOK-Pflegereport 2017 [28] über 40 % aller Heimbewohner:innen mit Antipsychotika behandelt. Viele Antipsychotika sind als potenziell inadäquate Medikamente für ältere Menschen in der PRISCUS-Liste [17] gelistet, da sie insbesondere bei älteren Menschen mit Demenz mit einem erhöhten Risiko für kardio- und zerebrovaskuläre Ereignisse, einer erhöhten Sterblichkeit sowie mit Nebenwirkungen, wie Müdigkeit, Sturz, Schwindel, extrapyramidalmotorischer Symptomatik und Beeinträchtigung der kognitiven Fähigkeiten verbunden sind [3, 9, 12, 14, 20–30]. Aus diesem Grund empfehlen Leitlinien zur Behandlung von Demenz, Antipsychotika nur bei speziellen Indikationen einzusetzen und – falls die Therapie mit Antipsychotika unumgänglich ist – die Antipsychotika möglichst kurz und in möglichst niedriger Dosierung zu verwenden [7, 11, 13, 15, 18, 23, 26]. In der deutschen S3-Leitlinie zur Behandlung von Demenz werden keine konkreten Empfehlungen zur Indikationsstellung und Vorgehensweise bei der Reduktion und dem Absetzen von Antipsychotika gegeben [11].

Auch ist der Einsatz von z. B. atypischen Antipsychotika zur Behandlung von BPSD bei Demenz auf Risperidon begrenzt, und dies gemäß Fachinformation auf eine Dauer von 6 Wochen.

In der Literatur gibt es trotz limitierter Datenlage Hinweise, dass ein Ausschleichen von Antipsychotika bei Demenz prinzipiell ohne Nachteil erfolgreich möglich ist [10, 29], wobei Langzeiteffekte auf Kognition und Psychomotorik nicht ausreichend untersucht wurden. Ausnahmen im Sinne erhöhter Rezidivraten scheinen Fälle, die besonders gut auf Antipsychotika wie Haloperidol oder Risperidon ansprechen, oder Betroffene mit deutlich ausgeprägten BPSD darzustellen [10, 29].

Um zu vermeiden, dass eine Behandlung mit Antipsychotika bei Menschen mit Demenz zu lange bzw. ohne entsprechende Indikation fortgesetzt wird, war unser Ziel, Handlungsempfehlungen zur Reduktion bzw. zum Ausschleichen von Antipsychotika zu entwickeln.

Die Handlungsempfehlungen sollen das Bewusstsein der Behandler:innen für die Notwendigkeit des „Deprescribings“ (Reduktion und Ausschleichen) schärfen und eine Hilfestellung diesbezüglich leisten.

## Methodik

Die Entwicklung der Handlungsempfehlungen erfolgte in einem mehrstufigen Prozess. In einem ersten Schritt wurde ein Entwurf anhand aktueller Literatur sowie den Ergebnissen der READY-Studie entwickelt.

Dieser Entwurf wurde in einem multiprofessionellen Expert:innengremium diskutiert und überarbeitet. Als Ergebnis wurden endgültige Empfehlungen zum Deprescribing formuliert. Diese Aussagen wurden schließlich in einem letzten Schritt im Rahmen einer Onlineumfrage von ärztlichen Demenzexpert:innen konsentiert.

### Literaturanalyse

Leitlinien zur Therapie von BPSD bei Demenz und zum Deprescribing von Antipsychotika wurden in den Datenbanken Medline, Embase, Scopus, PsycInfo, der AWMF-Leitlinien-Datenbank, der „guidelines international network library“ und den Internetauftritten von Fachgesellschaften recherchiert. Des Weiteren wurde die relevante Literatur zum Deprescribing von Antipsychotika bei Demenz über die oben erwähnten Datenbanken identifiziert.

### Erfahrungen aus einer eigenen Interventionsstudie

Die READY-Studie (Reduktion von Antipsychotika bei Heimbewohnern mit Demenz – eine Interventionsstudie, DRKS-ID: DRKS00017831) war eine Interventionsstudie, bei der eine Reduktion und ein Ausschleichen (Deprescribing) von Antipsychotika bei Menschen mit Demenz in Pflegeheimen durchgeführt wurde (s. Zusatzmaterial online, eAbb. 1). Ziel der Studie war es zu evaluieren, ob Deprescribing im klinischen Alltag in Pflegeheimen umsetzbar und durchführbar ist. Als Grundlage für das Vorgehen der Reduktion diente der in den kanadischen Leitlinien zum Deprescribing von Antipsychotika bei BPSD empfohlene Algorithmus von Bjerre et al. [7]. Den Heimärzt:innen wurde dabei jeweils ein individueller Reduktionsplan vorgeschlagen. Im Fall einer Anordnung des Reduktionsplans erfolgte eine Verlaufsbeobachtung während des Ausschleichens bzw. der Antipsychotikareduktion. Primärer Endpunkt war der Anteil der von den Heimärzt:innen angeordneten an den von den Studienärzt:innen vorgeschlagenen Reduktionsplänen. Sekundäre Endpunkte waren die Veränderung der BPSD, Kognition, Alltagskompetenz, medizinische Ereignisse, der psychopathologische Befund, körperliche und neurologische Parameter, Sturzereignisse, Mobilität sowie die allgemeine klinische Verbesserung während und nach der Reduktion bzw. dem Absetzen des Antipsychotikums. Zusätzlich wurden die Einstellungen der Angehörigen und behandelnden Ärzt:innen und der Eindruck der Pflegekräfte abgefragt.

Die Studie wurde von der Ethikkommission der Technischen Universität München genehmigt.

Da die Studie aufgrund der COVID-19-Pandemie im März 2020 gestoppt werden musste, erfolgte aufgrund der dadurch niedrigen Teilnehmerzahl lediglich eine qualitative Auswertung. Die Erfahrungen der Studienärztinnen mit dem Deprescribing von Antipsychotika wurden anhand eines Einzelinterviews aufgezeichnet und analysiert. Die Erkenntnisse aus der Studie sowie daraus resultierende Empfehlungen wurden in den ersten Entwurf der Handlungsempfehlungen aufgenommen.

### Multiprofessionelles Expert:innengremium

Auf Grundlage von sieben internationalen und nationalen Leitlinien [7, 11, 13, 15, 18, 23, 26], mehreren Studien [2, 4, 5, 9, 12, 14, 19, 20] und Reviews [1, 3, 6, 10, 16, 29] bzw. Metaanalysen [22, 25, 30] sowie den Erkenntnissen aus der READY-Studie wurden 51 Aussagen zum Deprescribing von Antipsychotika bei Demenz verfasst und einem multiprofessionellen Expert:innengremium vorgestellt sowie von diesem in einer Videokonferenz diskutiert und überarbeitet. Das Gremium bestand aus sieben Demenzexpert:innen: eine Pflegeheimleitung, eine psychiatrische Fachpflegekraft und Pflegedienstleitung, eine Heimärztin, eine Palliativmedizinerin, ein Psychiater, eine Psychiaterin und eine Neurologin. Die Pflegeheimleitung und die Heimärztin hatten bereits in der READY-Studie mitgewirkt, die anderen Teilnehmer:innen des Gremiums waren Mitarbeiter:innen an der Universitätsklinik der Technischen Universität München.

Im Rahmen einer Kommunikation per E‑Mail wurde schließlich in mehreren Durchgängen ein finaler Entwurf der Handlungsempfehlungen durch das multiprofessionelle Expert:innengremium konsentiert, der 32 Aussagen zum Deprescribing beinhaltete.

### Online Expert:innenumfrage

Die vom multiprofessionellen Expert:innengremium final zusammengestellten 32 Aussagen wurden schließlich in Form einer Onlineumfrage 59 ärztlichen Demenzexpert:innen aus Deutschland zur Stellungnahme und Konsensfindung vorgestellt. Die Expert:innen setzten sich zusammen aus Ärzt:innen, die in Kliniken (Universitätsklinika und Versorgungskrankenhäusern) im stationären oder ambulanten Bereich tätig waren sowie niedergelassenen Neurolog:innen, Psychiater:innen, Nervenärzt:innen oder Allgemeinärzt:innen. Die Expert:innen wurden persönlich per E‑Mail zur Teilnahme an der Umfrage eingeladen. 32 der 59 geladenen Expert:innen nahmen an der Umfrage teil (Tab. 1). Die Umfrage wurde pseudonymisiert durchgeführt, d. h. jede/jeder geladene Teilnehmer:in erhielt über eine nicht in die Auswertung involvierte Person (Treuhänderin) eine Pseudonymisierungsnummer. Die verwendete Software war „SoSci-Survey“. Die Expert:innen wurden aufgefordert anhand einer 5‑stelligen Likert-Skala (Tab. 2) ihre Zustimmung oder Ablehnung zu den jeweiligen Aussagen zu geben. Zusätzlich wurden demographische Daten der Teilnehmer:innen erhoben (Tab. 1). Am Ende der Umfrage ermöglichte ein Kommentarfeld schriftliche Anmerkungen. Die Durchführung der Onlineumfrage wurde durch die Ethikkommission der Medizinischen Fakultät der Technischen Universität München positiv votiert (#215/21 S‑EB).

**Table 1:** 

Geschlecht	*Männlich*	*Weiblich*	–	–
	20	12	–	–
Alter (Jahre)	*36–45*	*46–55*	*56–65*	*66–75*
	6	17	8	1
Fachbereich^a^	*Psychiatrie*	*Neurologie*	*Allgemeinmedizin*	*Geriatrie*
	23	15	1	8
Praxiserfahrung (Jahre)	*5–10*	*11–20*	*21–30*	*31–40*
	1	9	16	6
Anzahl Patient:innen mit Demenz/Monat	*5–10*	*10–30*	*30–50*	*>* *50*
	2	11	9	10
Einrichtung	*Krankenhaus/Klinik*	*Praxis*	–	–
	19	13	–	–

^a^Mehrfachangaben möglich

**Table 2:** 

1 = Stimme sehr zu
2 = Stimme zu
3 = Stimme weder zu noch nicht zu
4 = Stimme nicht zu
5 = Stimme überhaupt nicht zu
6 = Das kann ich nicht beurteilen

Die Auswertung der Onlineumfrage erfolgte anonymisiert über die Bestimmung des Prozentanteils der jeweiligen Antworten von „stimme sehr zu“ bis „stimme überhaupt nicht zu“ aus den Antworten aller Teilnehmer für die jeweilige Aussage. Dabei wurden die Antworten „stimme sehr zu“ (1) und „stimme zu“ (2) als „*Ja*“ zusammengefasst, die Antworten „stimme nicht zu“ (4) und „stimme überhaupt nicht zu“ (5) wurden als „*Nein*“ zusammengefasst. Ein Stimmenanteil von mehr als 75 % wurde als Konsens in Anlehnung an das Programm für nationale Versorgungsleitlinien definiert [8]. Aussagen, die einen positiven Konsens im Sinne einer Zustimmung erreichten, wurden entweder in den einleitenden Text der Handlungsempfehlungen integriert oder direkt als konkrete Handlungsempfehlung übernommen. Aussagen, die keinen Konsens erreichten oder mehrheitlich abgelehnt wurden, wurden verworfen.

## Ergebnisse

**Figure 1:**
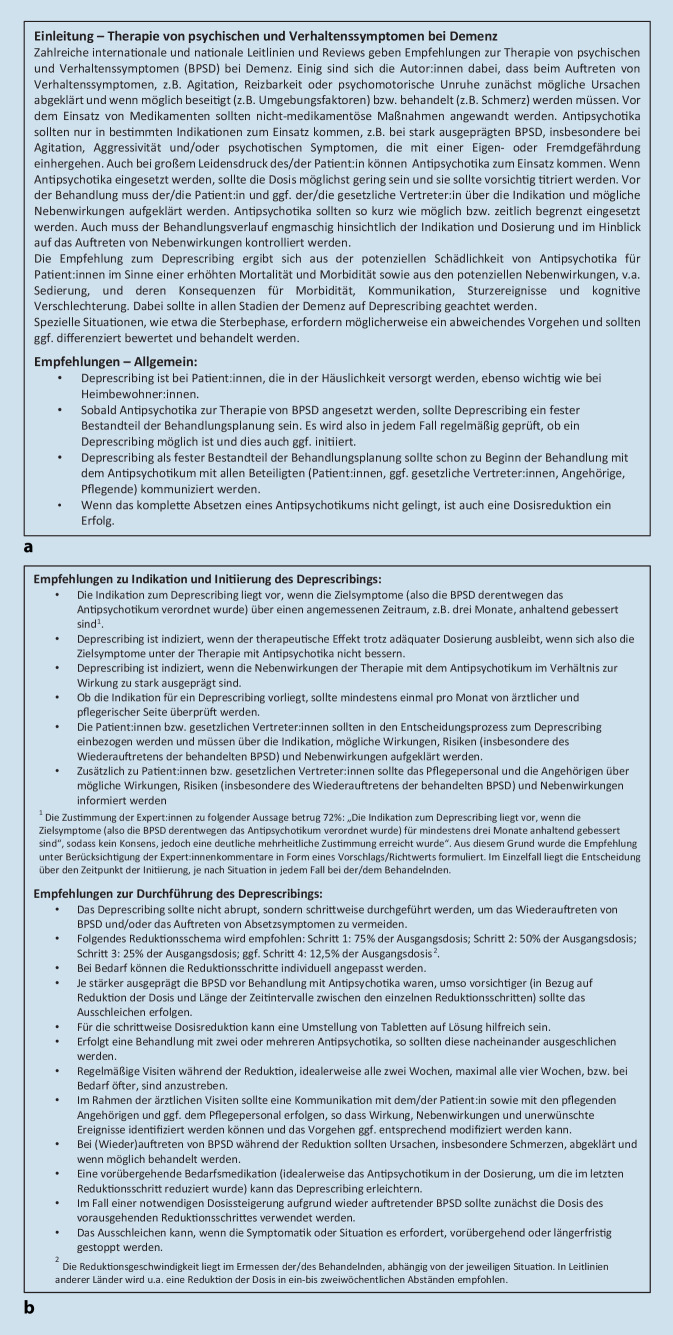

Vierundzwanzig der 32 in der Onlineumfrage präsentierten Aussagen zum Deprescribing von Antipsychotika bei Demenz erreichten einen zustimmenden Konsens von mehr als 75 %, davon 13 Aussagen einen starken Konsens von mehr als 95 %. Fünf Aussagen erreichten eine mehrheitliche Zustimmung (> 50 %), jedoch keinen Konsens. Bei einer Aussage lag die Zustimmung bei genau 50 %. Zwei Aussagen wurden mehrheitlich abgelehnt. Bei keiner Aussage lag ein negativer Konsens (> 75 % Nein-Stimmen) vor (siehe Zusatzmaterial online, eTab. 1). Einundzwanzig der konsentierten Aussagen wurden wörtlich als konkrete Empfehlungen übernommen, 3 Aussagen wurden in den einleitenden Text der Handlungsempfehlungen integriert (Abb. 1). Die 7 Aussagen, die keinen Konsens erreichten bzw. abgelehnt wurden, wurden nicht in die Handlungsempfehlungen aufgenommen. Die Aussage „Die Indikation für Deprescribing liegt vor, wenn die Zielsymptome (also die Symptome, aufgrund derer das Antipsychotikum verordnet wurde) für mindestens 3 Monate anhaltend gebessert sind“ erreichte 72 % Zustimmung. Sie wurde entsprechend den Kommentaren der Befragten in ihrer Formulierung verändert und mit einem Vermerk auf zugrunde liegende Leitlinien versehen. Eine weitere konsentierte Aussage wurde durch einen Vermerk auf zugrunde liegende Leitlinien ergänzt.

**Figure 2:**
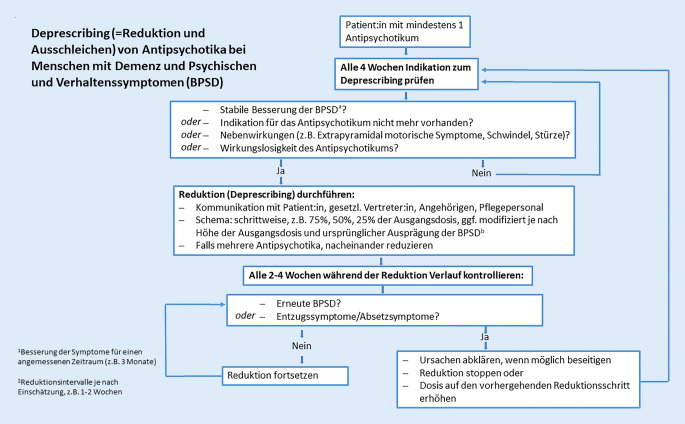

Neben dem einleitenden Text sind die einzelnen Aussagen der Handlungsempfehlungen in die Bereiche (1) Empfehlungen – Allgemeines, (2) Empfehlungen zu Indikation und Initiierung sowie (3) Empfehlungen zur Durchführung des Deprescribings aufgeteilt (Abb. 1). Ein Algorithmus „für die Kitteltasche“ fasst die wesentlichen Empfehlungen zusammen (s. Abb. 2).

## Diskussion

In einem mehrstufigen Prozess wurden Handlungsempfehlungen zum Deprescribing von Antipsychotika, die zur Behandlung von BPSD bei Demenz eingesetzt werden, entwickelt. Im letzten Schritt erfolgte eine Konsentierung durch 32 Demenzexpert:innen. Antipsychotika wie auch andere Psychopharmaka können bei BPSD und gerade in Krisen, z. B. bei Delirien, schon nach kurzer Zeit eine deutliche Linderung der Symptomatik bewirken. Der Dauergebrauch von Antipsychotika – ohne entsprechende medizinische Indikation – verbietet sich jedoch wegen der erhöhten Mortalität und Morbidität, die mit der Behandlung einhergehen, wie auch wegen der Nebenwirkungen, welche die Lebensqualität der Betroffenen deutlich einschränken können. Auch wenn die Datenlage limitiert ist (geringe Studienzahl, niedrige Patient:innenzahl, geringe Erfassung einzelner Parameter, kaum Daten zum Langzeitverlauf z. B. bez. Kognition) so zeigt sich doch in den dazu durchgeführten Studien, dass ein Absetzen bzw. Ausschleichen von Antipsychotika, die zur Behandlung von BPSD bei Demenz eingesetzt werden, insbesondere im Fall gering ausgeprägter Verhaltensauffälligkeiten gut möglich ist [10, 29]. Die Beendigung einer antipsychotischen Therapie ist beispielsweise mit Erfolgsraten von 73–90 % [10] nach einem Monat sowie von u. a. 69 % nach einem Jahr [5] verbunden und unterscheidet sich nicht signifikant von einer Therapiefortsetzung [10, 29]. Ausnahmen scheinen dabei Fälle mit einem primär guten Ansprechen auf Risperidon und Haloperidol sowie mit initial schweren BPSD darzustellen, bei denen ein Absetzen mit einer erhöhten Rückfallrate verbunden ist [10, 29]. Hingegen kann im Fall von gering ausgeprägten BPSD möglicherweise eine Verbesserung des Verhaltens erreicht werden [10]. Von Daher ist es Aufgabe der behandelnden Ärzt:innen, regelmäßig zusammen mit allen in die Behandlung und Pflege involvierten Personen und der/dem Betroffenen zu evaluieren, ob die Antipsychotikagabe noch indiziert ist oder ob die Medikamente ausgeschlichen werden können. Die Handlungsempfehlungen sollen hierfür das Bewusstsein schärfen und gleichzeitig eine Anleitung geben, wie ein Ausschleichen gut gelingen kann. Sie sind anwendbar im ambulanten und klinisch-stationären Setting ebenso wie in Einrichtungen der Langzeitpflege und richten sich an Ärzt:innen aller Fachrichtungen, die Menschen mit Demenz behandeln. Selbstverständlich besteht bei einer Reduktion von Antipsychotika immer das Risiko, dass behandelte BPSD wieder auftreten, insbesondere wenn diese vor der Behandlung deutlich ausgeprägt waren oder deutlich auf eine antipsychotische Behandlung angesprochen haben. Für diesen Fall empfehlen die Handlungsempfehlungen dann wieder eine entsprechende Aufdosierung der Medikamente. Aber selbst eine Dosisreduktion ist ein Erfolg, verglichen mit dem stoischen „weiter so“, das von einigen Behandler:innen verfolgt wird. Generell bedeutet das erfolgreiche Ausschleichen für jede:n einzelne:n Patient:in einen potenziellen Gewinn an Lebensqualität und Überlebenszeit.

Die Handlungsempfehlungen sind keine Leitlinien mit Empfehlungsgraduierung. Sie entbinden den/die Behandler:in nicht von eigenverantwortlichem und auf die entsprechende Behandlungssituation ausgerichtetem individuellem Handeln, vor allem auch im Hinblick auf die rechtlichen Rahmenbedingungen. Sie erheben keinen Anspruch auf Vollständigkeit. Auch beziehen sie sich nicht auf spezielle Fragestellungen, wie z. B. das Vorgehen in der Palliativsituation oder beim Vorhandensein von Komorbiditäten, wie etwa Schizophrenie oder schizoaffektiver Störung.

## Fazit für die Praxis

- Bei der Behandlung der psychischen und Verhaltenssymptome („Behavioral and Psychological Symptoms of Dementia“ [BPSD]) von Menschen mit Demenz mit Antipsychotika sollte stets und rechtzeitig Deprescribing (Reduktion und Ausschleichen) bedacht und, falls möglich, initiiert werden.
- Die Handlungsempfehlungen sollen das Bewusstsein für die Notwendigkeit zum Deprescribing von Antipsychotika schärfen und den behandelnden Ärzt:innen eine Hilfestellung bei der Indikationsstellung und Durchführung geben.
- Der Deprescribing-Algorithmus ist eine übersichtliche Zusammenfassung der Handlungsempfehlungen im Kitteltaschenformat.
- Bei den Empfehlungen handelt sich um Vorschläge, diese ersetzen jedoch nicht die individuelle Verantwortlichkeit des/der Behandler:in. Sie sind keine Leitlinien mit Empfehlungsgraduierung.
- Spezielle Fragestellungen z. B. Palliativsituationen oder Komorbiditäten wie Schizophrenie oder schizoaffektive Störung sind nicht Bestandteil der Empfehlungen und bedürfen einer gesonderten Einschätzung.
- Besondere Fälle mit potenziell hoher Rückfallrate, wie etwa stark ausgeprägte BPSD vor der antipsychotischen Behandlung oder exzellentes Ansprechen auf die antipsychotische Therapie, sind bei der Indikationsstellung und Durchführung zu beachten.

## Supplementary Information





