# Supplementary material for: Deprescribing of antipsychotic drugs for dementia: Recommendations for action on dose reduction and discontinuation attempts
Source: Nervenarzt. 2022 Jun 30;93(9):912–20. [Article in German] doi: 10.1007/s00115-022-01343-w (PMC9243982; doi:10.1007/s00115-022-01343-w)
Supplement: Supplementary file 1 [file 115_2022_1343_MOESM1_ESM.docx]

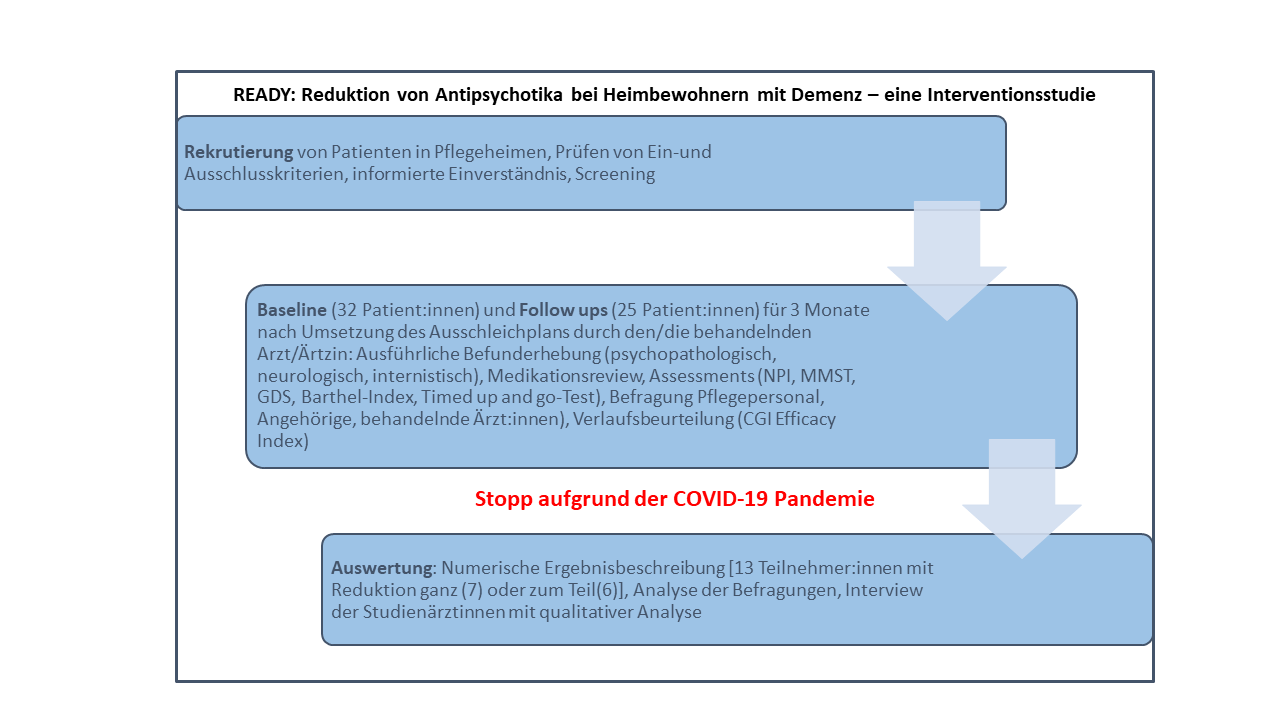


**eAbb. 1:** Aufbau und Inhalt der READY-Studie

NPI: Neuropsychiatric Interview; MMST: Mini-Mental-Status-Test; GDS: Geriatric depression scale; CGI: Clinical Global Impression
