# Supplementary material for: Deprescribing of antipsychotic drugs for dementia: Recommendations for action on dose reduction and discontinuation attempts
Source: Nervenarzt. 2022 Jun 30;93(9):912–20. [Article in German] doi: 10.1007/s00115-022-01343-w (PMC9243982; doi:10.1007/s00115-022-01343-w)
Supplement: Supplementary file 2 [file 115_2022_1343_MOESM2_ESM.docx]

**eTab.1:** Ergebnis der Onlineumfrage

| **Aussage** | **MW** | **SD** | **Med** | **Ja** | **Nein** | **W/N** |
| --- | --- | --- | --- | --- | --- | --- |
| 1. Die Empfehlung zum Deprescribing von Antipsychotika ergibt sich aus der potentiellen Schädlichkeit von Antipsychotika für Patient:innen im Sinne einer erhöhten Mortalität und Morbidität. | 1,38 | 0,49 | 1 | 100% | 0% | 0% |
| 1. Die Empfehlung zum Deprescribing ergibt sich auch aus den potentiellen Nebenwirkungen v.a. Sedierung und deren Konsequenzen für Mobilität, Kommunikation, Sturzereignisse und kognitive Verschlechterung. | 1,25 | 0,44 | 1 | 100% | 0% | 0% |
| 1. Deprescribing von Antipsychotika ist in allen Stadien der Demenz wichtig. | 1,53 | 0,76 | 1 | 91% | 3% | 6% |
| 1. Sobald Antipsychotika zur Therapie von BPSD angesetzt werden, sollte Deprescribing ein fester Bestandteil der Behandlungsplanung sein. Es wird also in jedem Fall regelmäßig geprüft, ob ein Deprescribing möglich ist und dies auch ggf. initiiert. | 1,25 | 0,44 | 1 | 100% | 0% | 0% |
| 1. Deprescribing als fester Bestandteil der Behandlungsplanung sollte schon zu Beginn der Behandlung mit dem Antipsychotikum mit allen Beteiligten (Patient:innen, ggf. gesetzliche Vertreter:innen, Angehörige, Pflegende) kommuniziert werden. | 1,69 | 0,78 | 2 | 88% | 3% | 9% |
| 1. Deprescribing ist bei Patient:innen, die in der Häuslichkeit versorgt werden, ebenso wichtig, wie bei Heimbewohner:innen. | 1,16 | 0,37 | 1 | 100% | 0% | 0% |
| 1. Die Indikation für Deprescribing liegt vor, wenn die Zielsymptome (also die BPSD, derentwegen das Antipsychotikum verordnet wurde) für mindestens drei Monate anhaltend gebessert sind. | 2,13 | 1,18 | 2 | 72% | 19% | 9% |
| 1. Deprescribing ist auch indiziert, wenn der therapeutische Effekt trotz adäquater Dosierung ausbleibt, wenn sich also die Zielsymptome unter der Therapie mit Antipsychotika nicht bessern. | 1,22 | 0,49 | 1 | 97% | 0% | 3% |
| 1. Deprescribing ist auch indiziert, wenn die Nebenwirkungen der Therapie mit dem Antipsychotikum im Verhältnis zur Wirkung zu stark ausgeprägt sind. | 1,22 | 0,49 | 1 | 97% | 0% | 3% |
| 1. Ob die Indikation für ein Deprescribing vorliegt, sollte regelmäßig, mindestens einmal pro Monat, von ärztlicher und pflegerischer Seite überprüft werden. | 1,72 | 0,96 | 1,5 | 88% | 6% | 6% |
| 1. Wenn die Antipsychotika zur Behandlung paranoid-halluzinatorischer Symptome („Wahn“, „Halluzinationen“) eingesetzt werden, sollte die Indikation zum Deprescribing zurückhaltend gestellt werden. | 3,34 | 1,00 | 4 | 28% | 53% | 19% |
| 1. Auf Deprescribing sollte verzichtet werden, wenn das Antipsychotikum zur Behandlung einer komorbiden Schizophrenie oder schizoaffektiven Störung eingesetzt wird. | 2,81 | 0,93 | 3 | 41% | 28% | 31% |
| 1. Das Deprescribing sollte nicht zu spät initiiert werden. Ein Zeitrahmen von sechs Monaten mit stabil gebesserter Zielsymptomatik sollte nicht überschritten werden. | 2,19 | 1,06 | 2 | 66% | 16% | 18% |
| 1. Wird ein Zeitrahmen von sechs Monaten überschritten, so ist die Gefahr für Absetzsymptome (z.B. Entzugserscheinungen) höher. | 3,47 | 1,02 | 3,5 | 16% | 50% | 34% |
| 1. Die Patient:innen bzw. gesetzliche Vertreter:innen sollten in den Entscheidungsprozess zum Deprescribing einbezogen werden und müssen über die Indikation, mögliche Wirkungen, Risiken (insbesondere des Wiederauftretens der behandelten BPSD) und Nebenwirkungen aufgeklärt werden. | 1,66 | 0,87 | 1 | 88% | 6% | 6% |
| 1. Zusätzlich zu Patient:innen bzw. gesetzlichen Vertreter:innen sollte das Pflegepersonal und die Angehörigen über mögliche Wirkungen, Risiken (insbesondere des Wiederauftretens der behandelten BPSD) und Nebenwirkungen informiert werden. | 1,38 | 0,55 | 1 | 97% | 0% | 3% |
| 1. Das Deprescribing sollte nicht abrupt, sondern schrittweise durchgeführt werden, um das Wiederauftreten von BPSD und/ oder das Auftreten von Absetzsymptomen zu vermeiden. | 1,22 | 0,42 | 1 | 100% | 0% | 0% |
| 1. Folgendes Reduktions-Schema wird empfohlen: Schritt 1: 75% der Ausgangsdosis; Schritt 2: 50% der Ausgangsdosis; Schritt 3: 25% der Ausgangsdosis; ggf. Schritt 4: 12,5% der Ausgangsdosis. | 2,31 | 1,09 | 2 | 81% | 9% | 10% |
| 1. Bei Bedarf können die Reduktionsschritte individuell angepasst werden. | 1,16 | 0,45 | 1 | 97% | 0% | 3% |
| 1. Für die schrittweise Dosisreduktion kann eine Umstellung von Tabletten auf Lösung hilfreich sein. | 1,72 | 0,85 | 1,5 | 81% | 3% | 16% |
| 1. Sollte ein Ausschleichen nicht möglich sein, weil Tabletten nicht weiter teilbar sind und das Präparat nicht als Lösung verfügbar ist, so sollte erwogen werden, ein Ausschleichen dadurch zu ermöglichen, dass das Antipsychotikum vorübergehend alle zwei Tage verabreicht wird. | 2,63 | 1,04 | 2 | 63% | 28% | 9% |
| 1. Die Zeitintervalle zwischen den einzelnen Reduktionsschritten sollten möglichst lang sein, es werden mindestens vier Wochen empfohlen. | 2,75 | 0,95 | 2,5 | 50% | 25% | 25% |
| 1. Je stärker ausgeprägt die BPSD vor Behandlung mit Antipsychotika waren, umso vorsichtiger (in Bezug auf Reduktion der Dosis und Länge der Zeitintervalle zwischen den einzelnen Reduktionsschritten) sollte das Ausschleichen erfolgen. | 1,75 | 0,84 | 2 | 88% | 6% | 6% |
| 1. Erfolgt eine Behandlung mit zwei oder mehreren Antipsychotika, so sollten diese nacheinander ausgeschlichen werden. | 1,56 | 0,62 | 1,5 | 94% | 0% | 6% |
| 1. Regelmäßige ärztliche Visiten während der Reduktion, idealerweise zweiwöchig, maximal vierwöchig, sind anzustreben bzw. bei Bedarf öfter. | 1,94 | 1,01 | 2 | 84% | 6% | 10% |
| 1. Im Rahmen ärztlicher Visiten sollten zusammen mit dem/der Patient:in und den Angehörigen bzw., falls vorhanden, auch dem Pflegepersonal Wirkung, Nebenwirkungen und unerwünschte Ereignisse identifiziert werden, so dass das Vorgehen ggf. entsprechend modifiziert werden kann. | 1,47 | 0,95 | 1 | 100% | 0% | 0% |
| 1. Für den Fall des Wiederauftretens von Symptomen während/ nach der Reduktion sollten zunächst nicht-medikamentöse Maßnahmen eingeleitet werden. | 2,41 | 1,04 | 2 | 56% | 19% | 25% |
| 1. Bei (Wieder)auftreten von BPSD während der Reduktion sollten Ursachen, insbesondere Schmerzen, abgeklärt und wenn möglich behandelt werden. | 1,41 | 0,56 | 1 | 97% | 0% | 3% |
| 1. Eine vorübergehende Bedarfsmedikation (idealerweise das Antipsychotikum in der Dosierung, um die im letzten Reduktionsschritt reduziert wurde) kann das Deprescribing erleichtern. | 1,59 | 0,71 | 1 | 88% | 0% | 12% |
| 1. Das Ausschleichen kann, wenn die Symptomatik oder Situation es erfordert, vorübergehend oder längerfristig gestoppt werden. | 1,31 | 0,47 | 1 | 100% | 0% | 0% |
| 1. Im Fall einer notwendigen Dosissteigerung aufgrund wieder auftretender BPSD sollte zunächst die Dosis des vorausgehenden Reduktionsschrittes verwendet werden. | 1,91 | 0,69 | 2 | 88% | 3% | 9% |
| 1. Wenn das komplette Absetzen eines Antipsychotikums nicht gelingt, ist auch eine Dosisreduktion ein Erfolg! | 1,25 | 0,62 | 1 | 97% | 3% | 0% |

Dargestellt ist jeweils der Mittelwert aus den 5 Antwortmöglichkeiten der Likert-Skala (MW), die Standardabweichung (SD), der Median (Med) sowie der Prozentanteil an Ja-Stimmen (1 und 2 der Skala: stimme sehr zu/stimme zu), der Nein-Stimmen (4 und 5: stimme nicht zu/stimme überhaupt nicht zu) und W/N-Stimmen (3: stimme weder zu noch nicht zu), im Fall der Antwort 6 (das kann ich nicht beurteilen), was bei insgesamt 2 Aussagen, jeweils einmal der Fall war, wurde dieser Teilnehmer nicht in die Auswertung einbezogen.

Die Aussagen 11 und 14 wurden mehrheitlich abgelehnt, die Aussagen 12, 13, 21, 22 und 27 (alle grau unterlegt) erreichten eine mehrheitliche Zustimmung, jedoch keinen Konsens, so dass diese Aussagen nicht in die Handlungsempfehlungen aufgenommen wurden.

Die Aussage 7 (hellblau unterlegt) verfehlte knapp einen Konsens mit 72% Ja-Stimmen. Sie wurde unter Berücksichtigung der Kommentare in der Formulierung angepasst und mit einem Hinweis auf Empfehlungen aus zugrundeliegenden Leitlinien versehen.
